# Highly Stable Spatio-Temporal Prediction Network of Wavefront Sensor Slopes in Adaptive Optics

**DOI:** 10.3390/s23229260

**Published:** 2023-11-18

**Authors:** Ning Wang, Licheng Zhu, Qiang Yuan, Xinlan Ge, Zeyu Gao, Shuai Wang, Ping Yang

**Affiliations:** 1 National Key Laboratory of Optical Field Manipulation Science and Technology, Chinese Academy of Sciences, Chengdu 610209, China; w_ning@163.com (N.W.); xinlan_ge@163.com (X.G.); gaozeyu1994@hotmail.com (Z.G.); wangshuai@ioe.ac.cn (S.W.); 2 Key Laboratory on Adaptive Optics, Institute of Optics and Electronics, Chinese Academy of Sciences, Chengdu 610209, China; 3 Institute of Optics and Electronics, Chinese Academy of Sciences, Chengdu 610209, China; 4 School of Electronic, Electrical and Communication Engineering, University of Chinese Academy of Sciences, Beijing 100049, China; 5 Facility Design and Instrumentation Institute, China Aerodynamics Research and Development Center, Mianyang 621000, China; yqcardc@163.com

**Keywords:** adaptive optics, wavefront prediction, deep learning, laser atmospheric transmission

## Abstract

Adaptive Optics (AO) technology is an effective means to compensate for wavefront distortion, but its inherent delay error will cause the compensation wavefront on the deformable mirror (DM) to lag behind the changes in the distorted wavefront. Especially when the change in the wavefront is higher than the Shack–Hartmann wavefront sensor (SHWS) sampling frequency, the multi-frame delay will seriously limit its correction performance. In this paper, a highly stable AO prediction network based on deep learning is proposed, which only uses 10 frames of prior wavefront information to obtain high-stability and high-precision open-loop predicted slopes for the next six frames. The simulation results under various distortion intensities show that the prediction accuracy of six frames decreases by no more than 15%, and the experimental results also verify that the open-loop correction accuracy of our proposed method under the sampling frequency of 500 Hz is better than that of the traditional non-predicted method under 1000 Hz.

## 1. Introduction

AO technology is an effective means to compensate dynamic wavefront distortion. However, the actual AO system is limited by the read-out data delay of the SHWS and the control calculation delay, it usually has a correction delay of at least 2–3 sampling cycles [1,2,3]. As a result, SHWS usually needs to greatly increase the sampling frequency to cope with the high time-frequency wavefront distortion. This has a very high requirement for hardware processing speed, which greatly increases the system cost. At the same time, high frame frequency sampling will inevitably shorten the exposure time, which has adverse effects on the detection of dim targets. However, if the sampling frequency decreases, the compensated wavefront on DM will lag behind the actual wavefront, and the multi-frame delay error will seriously affect the correction performance of AO system [4,5]. Therefore, it is of great research significance and application value to obtain multi-frame correction results with higher accuracy and stronger stability under the condition that the SHWS sampling frequency is lower than the time variation frequency of atmospheric turbulence (f_SHWS_ < f_ATurb_).

Forward prediction correction technology can effectively alleviate the problem of multi-frame delay by predicting the future wavefront correction information, such as the more classical recursive least squares (RLS) control algorithm [6,7,8,9] and Kalman filter-based linear quadratic Gaussian (LQG) control algorithm [10,11,12,13,14,15]. Especially in recent years, neural network (NN) methods have shown unique advantages in AO prediction correction due to their strong nonlinear fitting ability, anti-interference robustness and generalization ability [16,17]. The relevant research on the single-frame output prediction model focuses on the improvement of prediction accuracy [18,19,20,21,22,23,24], and does not fully consider the problem of the number of specific multiple delay frames in the actual AO system. When dealing with the distortion of time frequency higher than the SHWS sampling frequency, the multi-frame delay in the AO system cannot be effectively compensated. And so, Swanson et al., 2018 [25] demonstrated their ability to predict up to five future wavefronts with 1 nm RMS wavefront error by using long short-term memory (LSTM) networks. Chen et al., 2021 [22] used a classical U-Net convolutional NN architecture to predict the future eight frames under 32 prior wavefronts of the input history.

However, the above multi-frame prediction methods show that with an increase in the number of predicted frames, the residual wavefront root mean square (RMS) value synchronously increases, the prediction accuracy decreases rapidly, and then the correction performance of the multi-frame delay system is significantly reduced. Based on the above description, we propose a spatio-temporal prediction network based on deep learning to obtain a high-stability and high-precision multi-frame prediction output. In our proposed spatio-temporal prediction network, we mainly include an attention mechanism and a residual learning and convolutional LSTM (ConvLSTM) module. The attention mechanism and residual learning module are used to extract the spatial features of the multi-frame prior input wavefront. The ConvLSTM network is used to gradually update the wavefront input to focus on the temporal features, and the final multi-frame prediction results are obtained. We show that at the condition of f_SHWS_ < f_ATurb_, our proposed network can obtain high-stability and high-precision open-loop wavefront slope of six frames in the future by using only 10 frames prior information, and verify the effectiveness and stability of our proposed network by using the experimental open-loop data collected by a 1 km laser atmospheric transmission system.

## 2. Methodology

**Figure 1:**
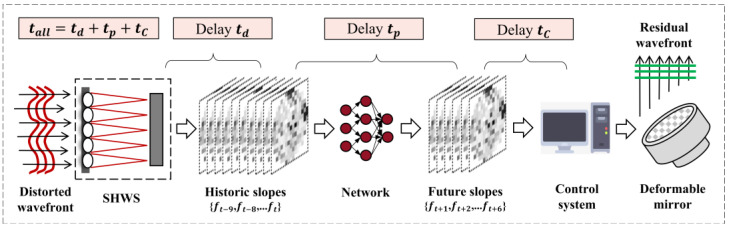
Schematic diagram of AO open-loop prediction correction system based on the distorted wavefront of atmospheric turbulence.

In conventional non-predicted AO systems, DM compensates for current wavefront distortion directly by loading historical wavefront information [3]. In order to improve the real-time correction capability, we adopt a deep learning-based method for multi-frame wavefront prediction to adapt to different delay errors at the condition of f_SHWS_ < f_ATurb_. The schematic diagram of an AO open-loop prediction correction system based on the distorted wavefront of atmospheric turbulence is shown in Figure 1. The AO system first uses SHWS to detect the wavefront aberration of atmospheric turbulence. Then, the NN prediction model predicts the wavefront aberration of the future time by taking the historical wavefront slope data as input, and the controller converts the predicted slope into the DM control voltage. Assuming that the time delay calculated by SHWS wavefront detection, NN prediction and wavefront controller processing is *t_d_*, *t_p_* and *t_c_*, respectively, then the total time error from the detection of wavefront aberration to the completion of phase correction are the sum of the three. If the NN can predict sufficient and accurate future slopes, then the corrected wavefront loaded on the DM is the true distorted wavefront currently detected, and real-time compensation can be completed.

### 2.1. Atmospheric Turbulence Data Set Generation

The atmospheric frozen flow hypothesis is a widely used modeling method for atmospheric turbulence [26,27,28,29]. In the simulation, we simulated a 1 km near-surface near-horizontal laser atmospheric transmission observation system based on Kolmogorov turbulence statistical theory [30], with a transmitting/receiving height of about 10 m. Five sets of atmospheric turbulence training dataset with different turbulence intensity were simulated according to the HV-57 refractive index structure constant model to improve the generalization ability of the network. The experimental measurements show that the variation in refractive index structure constant ($C_{n}^{2}$) at the same height in the four seasons in the region where our experimental system is located is generally about an order of magnitude. Therefore, five values within an order of magnitude of $C_{n}^{2}$ calculated under simulation conditions were considered in the simulation to simulate five different atmospheric turbulence intensity, in which $C_{n}^{2}$ and wind speed of each set of data were calculated by the relationship between atmospheric coherence length $r_{0}$ and $C_{n}^{2}$, and the Buffton wind speed model [24]. The phase screen of atmospheric turbulence is generated by Fourier series method through time evolution, and each phase screen contains a turbulence structure of 5 layers.

The sampling frequency is set to 1000 Hz to facilitate comparison of subsequent high frame frequency sampling open-loop correction. During training, frame extraction is used to perform prediction under the condition of 500 Hz sampling frequency, then in an AO system with a delay of 2–3 sampling cycles, the delay frame number may be as high as 4–6 frames. Under the condition of the strongest simulated turbulence intensity, we conducted a comparative test of the average RMSe of 200 test sets data by inputting 6–12 frames. We found that the prediction performance remained basically unchanged when the input exceeded 10 frames and beyond. Considering the problem that more wavefront input would increase the calculation and time consumption, we predict the uncorrected slope of the next 6 frames based on the historical 10 frames wavefront slope sequence of SHWS detection under the open-loop conditions. The telescope aperture is set to 1 m, the spatial sampling number is 256 × 256 pixels, the wavelength is 1064 nm, the lens let array of SHWS is 16 × 16, the sampling frequency is 500 Hz, and the wind direction is randomly 0–360°.

**Figure 2:**
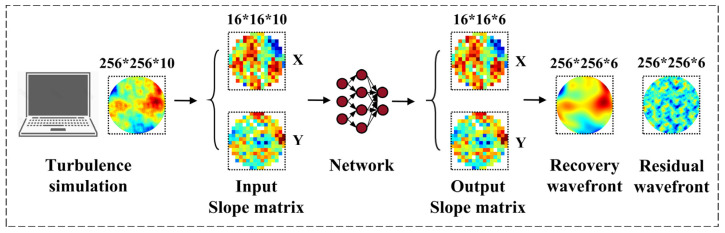
Schematic diagram of input and output settings for network model training.

Different *D*/*r*_0_ represent different turbulence intensities. We chose 10,000 sets of slope datasets for each of the five different *D*/*r*_0_ to train our model, so as to improve the generalization ability of the network. The dataset is divided into training and testing dataset in a 4:1 ratio. Specifically in this paper, for the images in the dataset, the first 10 frames are taken as the input, and frames 11–16 are taken as the label of the network to form a training pair. In the atmospheric simulation, we simulated the wavefront phase, but in the network model training, we selected the wavefront slope data, which is more easily obtained in the actual AO system and has a smaller dimension; that is, the input for each time is the centroid displacement in the x and y directions of the SHWS subaperture array, the input dimension is 16 × 16 × 2 × 10, and the output dimension is 16 × 16 × 2 × 6. The input/output settings are shown in Figure 2, in which recovery wavefronts are used as true wavefront for subsequent predictive performance tests.

### 2.2. Network Model Settings

**Figure 3:**
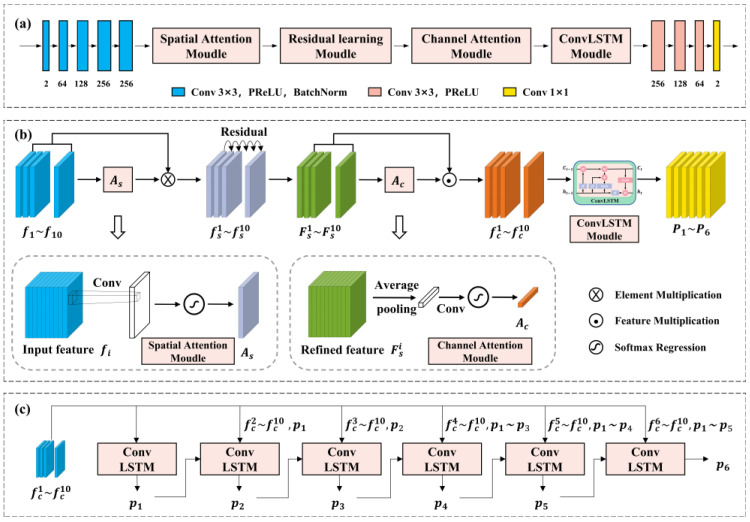
Our proposed spatio-temporal prediction network. (**a**) The overall architecture we propose; (**b**) the detailed structure we propose; (**c**) the ConvLSTM stepwise output mode.

In this paper, we proposed a spatio-temporal prediction network based on deep learning, and the network structure diagram is shown in Figure 3. The network first uses the spatial attention mechanism to pay attention to similar target features in each frame of distorted wavefront, and assigns different weights to each element in each frame. As shown in the figure, $f_{s}^{i}$ is the weighted characteristic wavefront, which well summarizes the similar target features in the continuous distorted wavefront. However, with the deepening of the number of network layers, the problem of gradient disappearance of the objective function may occur, which means the network parameters of the principle output layer cannot be effectively learned. The residual learning network can effectively alleviate it, improve the depth of the training network, and refine similar features between two consecutive frames and eliminate redundant information to obtain higher prediction accuracy, which has been verified in previous work [24]. Therefore, we use residual learning strategy to refine similar features for structural feature extraction.

In addition, although the distorted wavefront of each frame input contains target features, these features contribute differently to the obtained final prediction results. Similarly to the spatial attention mechanism, we use the channel attention mechanism to focus on those distorted wavefront that contain more target features, rather than treating the features from each frame equally. Finally, in order to make the multi-frame output have better stability, the extracted features are successively passed through the ConvLSTM network with time series features [31] to obtain the final prediction results. The training process of this paper is different from the traditional training methods; that is, the input prior does not necessarily come from all the true wavefront. It is a gradually updated process; that is, the specific training process is frame 1–10 to output frame 11, the second round of input is 2–10 combined with the last predicted frame 11 to output frame 12, the third round of input is 3–10 combined with frames 11 and 12 to output frame 13… and so on, as shown in Figure 3c.

In our proposed model, the L1_Loss function is used to minimize the sum of all absolute value errors between the true value and the predicted value. As shown in Equation (1):

(1) $$L1\_loss_{k}=\frac{\sum_{i=1}^{n}\left|y{\left(i\right)}_{k\_predict}-y{\left(i\right)}_{k\_true}\right|}{n}\;k=(1,2,3...6)$$

where *n* is all the elements. $y{\left(i\right)}_{k\_predict}$ is the predicted value of the distorted wavefront of the k-frame, and $y{\left(i\right)}_{k\_true}$ is the true value. The total function of this model is:

(2) $$Loss=\frac{\sum_{k=1}^{10}L1\_Loss_{k}}{6}$$

## 3. Results and Discussion

### 3.1. Simulation Data Analysis

After selecting the optimal model, we verify the accuracy and stability of our proposed model through the following two sets of test methods. All of the following prediction network datasets are sampled at a frequency of 500 Hz. In order to compare our proposed method with open-loop compensation under non-predicted conditions with high frame frequency sampling, we add a set of 1000 Hz to the non-predicted method. The non-predicted methods of 500 Hz and 1000 Hz specifically refer to the direct use of historical wavefront phase to compensate the current wavefront phase at their respective sampling frequencies. For example, in a two-frame delay system, the wavefront at the k-2 moment is used to compensate for the distorted wavefront at the k moment. In a three-frame delay system, the wavefront at the k-3 moment is used to compensate, and so on. The spatial prediction network model refers to the combination of classical CNN and spatial attention mechanism. The time prediction model refers to the classical ConvLSTM network. The training sets of the above two prediction networks are completely consistent with the training dataset of our proposed model. For all of the tests below, our results are based on averages of 200 test sets in order to achieve more realistic and accurate predicted results. We used two parameters to evaluate the accuracy of the prediction methods:

i. RMSe: Residual wavefront RMS;
ii. SSIM: Structural similarity index.

(1) The generalization ability is tested by using the distorted wavefront data sets of different *D*/*r*_0_, namely different atmospheric turbulence intensities, which have never been trained by the network.

(2) Under the atmospheric turbulence condition of *D*/*r_0_* = 26.32, the performance of our proposed method, the time/spatial prediction network, the 500 Hz non-predicted method, and the 1000 Hz non-predicted method is compared and analyzed using the same test dataset.

**Figure 4:**
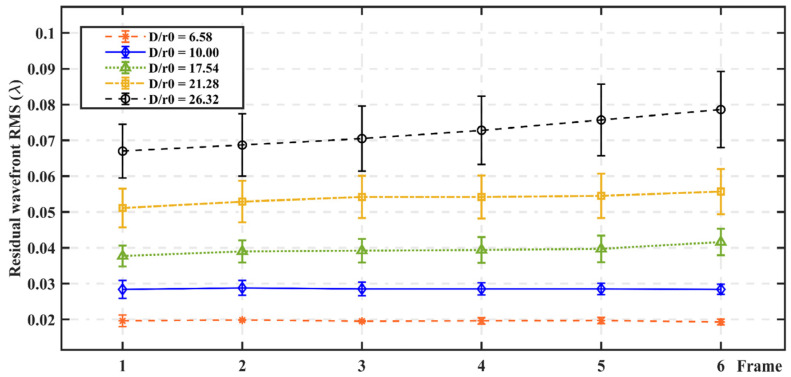
RMSe of 6 consecutive frames of distorted wavefront under different *D*/*r_0_*.

In test group (1), although the atmospheric turbulence distorted wavefront data of the test dataset is not part of the training dataset, has never been recognized by the network, and is separated from the training dataset distribution by 2000 frames, the parameters such as wind vectors in each test dataset are consistent with the training dataset at the same atmospheric turbulence intensity. Therefore, in this case, the predictor is expected to achieve its best performance. As shown in Figure 4, under the same delay condition, with an increase in *D*/*r*_0_, the RMSe of the six consecutive frames of distorted wavefront increases gradually. As the number of delayed frames increases, the larger the *D*/*r*_0_ is, the more unstable the predicted performance.

**Figure 5:**
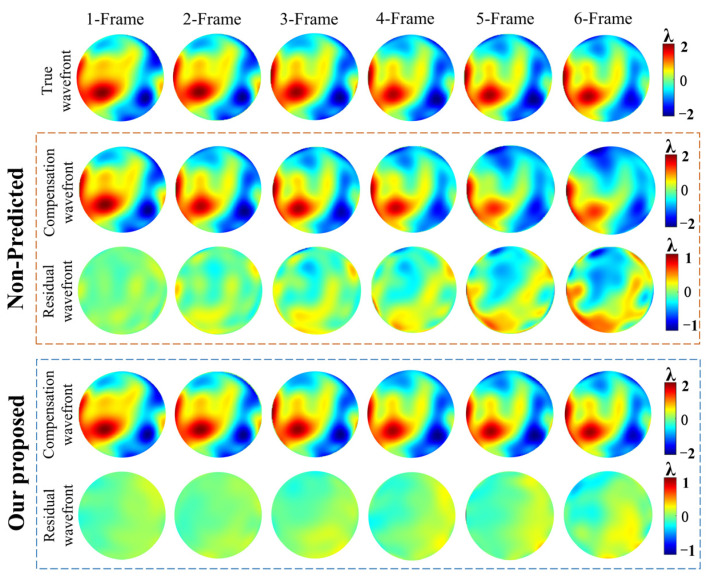
Continuous 6 frames of true distorted wavefront, open-loop compensation wavefront and residual wavefront of non-predicted method and our proposed prediction method. (*D*/*r_0_* = 26.32).

At the sampling frequency of 500 Hz, compared with the non-predicted methods, our proposed prediction network shows obvious open-loop compensation advantages. As shown in Figure 5, we show the compensation results of our proposed method and non-predicted method for random consecutive 6 frames of true wavefront under the condition of 1–6 frame delay, respectively.

**Figure 6:**
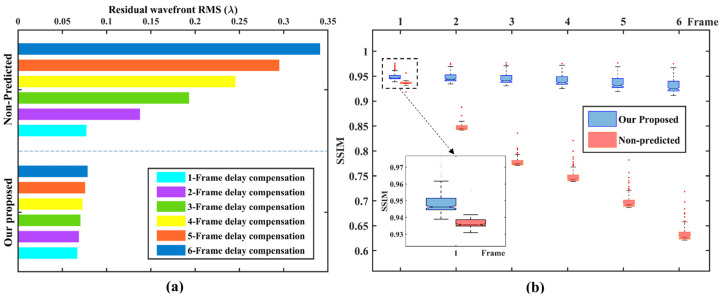
Open-loop compensation results under different delay conditions. (*D*/*r*_0_ = 26.32). (**a**) RMSe comparison results of non-predicted method and our proposed method; (**b**) SSIM comparison results of non-predicted method and our proposed method.

For a quantitative and more intuitive representation, we show the average compensation results for 100 sets of six frames consecutive true wavefront, as shown in Figure 6. In Figure 6a, it can be seen that with an increase in the number of delay frames, the prediction accuracy of our proposed method gradually decreases, but compared with non-predicted methods, it still shows higher prediction stability. When the number of delayed frames is 2, the prediction accuracy is improved by 50.2% compared to the non-predicted method; when the number of delayed frames is 4, the prediction accuracy is improved by 70.3%; and when the number of delayed frames is 6, the prediction accuracy is improved by 77%. It is proved that our proposed prediction network is effective in the actual AO delay system, and the system with more delay frames has greater performance improvement. In Figure 6b, it can be seen that as the number of delayed frames increases, the SSIM value of non-predicted methods decreases rapidly, while the SSIM of our proposed method remains stable above 0.9, which further verifies the prediction accuracy and stability of our proposed method.

**Figure 7:**
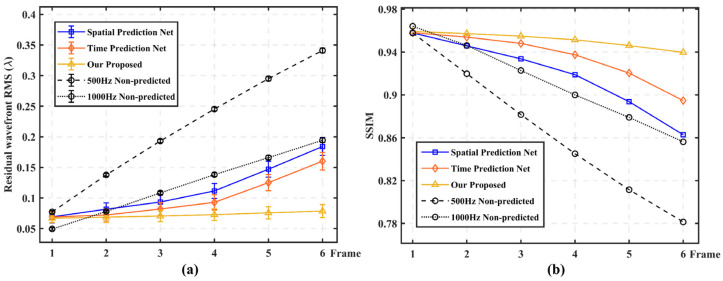
Prediction performance comparison of our proposed network with 500 Hz non-predicted, 1000 Hz non-predicted, spatial prediction and temporal prediction models (simulation data). (**a**) RMSe comparison of the five methods; (**b**) SSIM comparison of the five methods.

In test group (2), in order to further demonstrate the prediction accuracy and stability of our proposed method, we conducted four sets of comparison experiments under the turbulence intensity of *D*/*r*_0_ = 26.32. Figure 7 shows that under different multi-frame delay conditions, the high frame frequency sampling method (1000 Hz non-predicted) and the three prediction networks all have smaller RMSe and higher SSIM compared with the 500 Hz non-predicted method. Under the 2–6 frame delay condition, our proposed method all show the best prediction performance, but when the delay frame number is 1 frame, the 1000 Hz non-predicted method performs better. But at this time, the sampling frequency is consistent with the change frequency of distorted wavefront, and the system delay is at least 2–3 frames, so this advantage is not prominent. In addition, it can be seen that it is difficult to ensure the stability of continuous multi-frame prediction accuracy, whether it is high frame frequency sampling or only considering the correlation of wavefront distortion in time or space.

When the sampling frequency is 500 Hz, the system delay is about 4–6 frames. Therefore, we quantitatively demonstrate the performance improvement of our proposed prediction method, spatial prediction network and time prediction network compared with 500 Hz non-predicted method and 1000 Hz non-predicted method, respectively, under the delay conditions of four-frame and six-frame. As shown in Table 1, it can be seen that our proposed method has more obvious compensation advantages. Moreover, the prediction accuracy based on RMSe of the proposed for the next six frames of wavefront decreases by no more than 15%, which shows a high prediction stability.

In our proposed prediction model, the attention mechanism module and the residual learning module focus on finely extracting the spatial structure between the continuous wavefront. ConvLSTM focuses on the temporal correlation, and the output result of each time is used as the input for the next time. Although this training method of gradual output will bring a certain error accumulation, and the prediction accuracy will gradually decrease as the time goes on, but the output result of each frame comes from 10 consecutive frames of a priori including the most recent one, so the more accurate the prediction outputs of the previous frames are, the more accurate the later ones will be, which largely ensures the high stability of the output.

### 3.2. Experimental Data Verification

**Figure 8:**
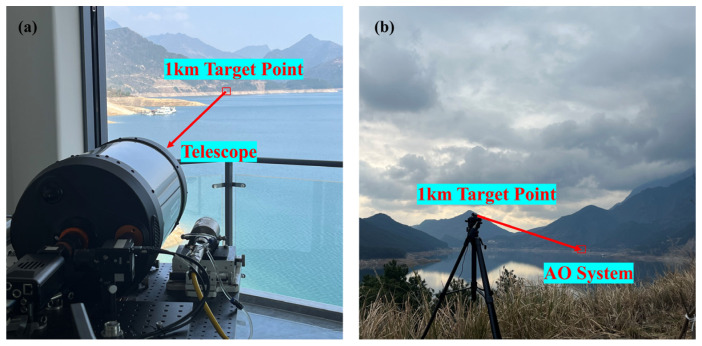
Schematic diagram of a 1 km laser atmospheric transmission system. (**a**) The perspective of the receiving end (AO system). (**b**) The perspective of the transmitting end (target pure cooperative target light source).

The performance of our proposed method is also verified in the experimental data, we tested one-time open-loop data collected from an actual 1 km laser atmospheric transmission system. The laser light source in the system is a purely cooperative target, as shown in Figure 8.

We collected a set of SHWS data with *D*/*r_0_ =* 18.7, a sampling frequency of 1000 Hz, and an acquisition time of 10 s. Table 2 shows the parameters of the cooperative target and SHWS in the experiment, as well as some parameters for estimating atmospheric conditions.

**Figure 9:**
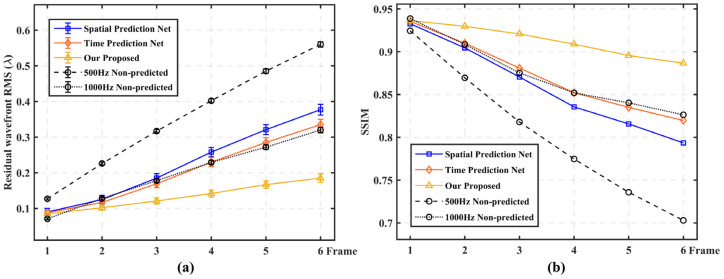
Prediction performance comparison of our proposed network with 500 Hz non-predicted, 1000 Hz non-predicted, spatial prediction and temporal prediction models (experimental data). (**a**) RMSe comparison of the five methods; (**b**) SSIM comparison of the five methods.

The result display of experimental data is based on the model after fine-tuning the simulation model; in other words, the experimental data is extracted at a sampling frame frequency of 500 Hz and then trained again on the basis of this model. This is because there is a large degree of difference between the simulated data and the experimental data in essence. The simulated data is a frozen and discrete noise-free model, while the experimental data has more uncertain errors, such as non-stationary characteristics of wind speeds and wind directions. However, the fine-tuning training is very fast, because our proposed model has learned the law of feature extraction and successfully identified the frozen flow part of the experimental data. In the fine-tuning process, we directly use the collected SHWS slope data as the training set data, and we reserve 500 frames of data as the test dataset and do not participate in the training process, so the test dataset has not been trained in advance. When analyzing the prediction performance, we carried out 65-order Zernike mode wavefront restoration processing on the SHWS label and the prediction data. Since tilt aberration had been removed in the obtained experimental data, which was only aimed at the wavefront signal driving the deformation of the DM. Figure 9 shows the prediction performance of our proposed method with non-predicted, spatial prediction and time prediction network.

Experimental test results show that under the different multi-frame delay conditions, the high frame frequency sampling method and the three prediction networks still have smaller RMSe and higher SSIM compared with the 500 Hz non-prediction method. The difference between the simulation results is that the high frame frequency sampling method is superior to the space/time prediction model. However, our proposed method still maintains a relatively high stability output. This is because the spatial and temporal similarity between the actual adjacent wavefront is smaller than the simulation data due to the influence of changing wind speed, for instance, which once again proves that a single capture time/space correlation is difficult to better satisfy the distortion compensation under real conditions. Similarly, for example, under the delay conditions of four-frame and six-frame, respectively, the average RMSe of our proposed model is 0.142λ and 0.185λ, respectively, compared with the 500 Hz non-predicted method, the compensation accuracy is improved by 64.7% and 67.0%, respectively. Compared with 1000 Hz non-predictive method, it is improved by 38.0% and 42.2%. However, in the case of our proposed method, this stability decreases compared to the simulation dataset test results. We attribute this performance degradation to more constantly changing factors in the real environment, such as ambient light intensity, wind speed, humidity, etc. More in-depth exploration will be carried out in our follow-up work.

**Figure 10:**
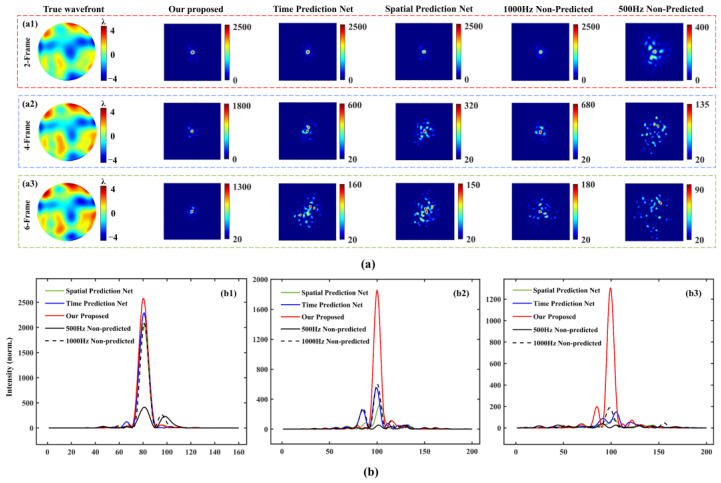
Far-field with open-loop compensation under 2-frame, 4-frame and 6-frame delay conditions. (**a**) The far-field after open-loop compensation of our proposed, 500 Hz non-predicted method, 1000 Hz non-predicted method, spatial prediction and time prediction model under 2-frame (**a1**), 4-frame (**a2**) and 6-frame (**a3**) delay conditions. (**b**) Intensity distribution of the far-field center horizontal line after open-loop compensation of our proposed, 500 Hz non-predicted method, 1000 Hz non-predicted method, spatial prediction and time prediction model under 2-frame (**b1**), 4-frame (**b2**) and 6-frame (**b3**) delay conditions.

Furthermore, we recorded the far-field of the true distorted wavefront to be corrected under the conditions of two-frame, four-frame and six-frame delay by using our proposed method, 500 Hz non-predicted method, 1000 Hz non-predicted method, spatial prediction and time prediction network. As can be seen from Figure 10a,b, regardless of the delay condition, the energy of our proposed method is more concentrated after open-loop compensation. From which it can be found that under the delay conditions of two-frame, four-frame and six-frame, the maximum intensity of the far-field focus of our proposed is about 6.2 times, 13.8 times and 14.1 times of the 500 Hz non-predicted method, and 1.2 times, 2.7 times and 6.9 times of the 1000 Hz non-predicted method after open-loop correction, respectively. It is quantitatively proved that our proposed prediction method can obtain better open-loop correction results under the condition of low sampling frequency than the traditional non-predicted method with high frame frequency sampling.

## 4. Conclusions

In this paper, a multi-frame wavefront prediction network is proposed, which takes into account the spatio-temporal coupling characteristics of the atmosphere. The network uses an attention mechanism and residual learning module to extract spatial features, and a ConvLSTM network to focus on the time series features by gradually updating the input training mode, and obtains the final multi-frame prediction results with high precision and high stability. At the same time, the model can predict the future multi-frame wavefront with relatively less prior wavefront input. The simulation results under various distortion intensities show that the prediction accuracy of six frames decreases by no more than 15%. The experimental results also confirm that under the condition of a sampling frame frequency of 500 Hz and a delay of six frames, the maximum intensity of far-field focus after open-loop correction is about 6.9 times that of 1000 Hz traditional non-predicted method. In summary, the results show that when the sampling frequency of SHWS is lower than the frequency of wavefront aberration change, our proposed prediction method can achieve better correction effect than the traditional non-predicted method in the low frame frequency sampling AO system, and can ensure sufficient exposure time.

## Figures and Tables

**Table 1:** Compared with 500 Hz non-predicted and 1000 Hz non-predicted methods, the compensation accuracy increase percentage of the three prediction methods.

	Methods	4-Frame Delay	6-Frame Delay
500 Hz Non-predicted	Our proposed	70.0%	76.8%
	Spatial Prediction Net	54.3%	62.0%
	Time Prediction Net	46.0%	53.1%
1000 Hz Non-predicted	Our proposed	47.1%	59.3%
	Spatial Prediction Net	18.8%	32.6%
	Time Prediction Net	5.2%	17.5%

**Table 2:** Parameters related to 1 km laser atmospheric transmission system.

Simulation Parameters	Values
Diameter	0.28 m
SHWS lens let array	16 × 16
CCD	256 × 256 pixels
Wavelength	1064 nm
*r*_0_	1.49 cm
Wind Speeds	3.0–5.5 m/s
Wind Directions	R [0–360°]
Transmit/Receive Altitude	10 m
Transmission Distance	1 km

## Data Availability

The data presented in this study are available on request from the corresponding author. The data are not publicly available for privacy reasons.
